# Structural Features Affecting the Interactions and Transportability of LAT1-Targeted Phenylalanine Drug Conjugates

**DOI:** 10.1021/acs.molpharmaceut.2c00594

**Published:** 2022-11-17

**Authors:** Katayun Bahrami, Juulia Järvinen, Tuomo Laitinen, Mika Reinisalo, Paavo Honkakoski, Antti Poso, Kristiina M. Huttunen, Jarkko Rautio

**Affiliations:** School of Pharmacy, University of Eastern Finland, P.O. Box 1627, Kuopio FI-70211, Finland

**Keywords:** LAT1, l-type amino acid transporter 1, IFD, induced-fit docking, QSAR, quantitative structure−activity relationship, HEK-hLAT1, human embryonic kidney cell line inducible for human l-type amino acid transporter 1

## Abstract

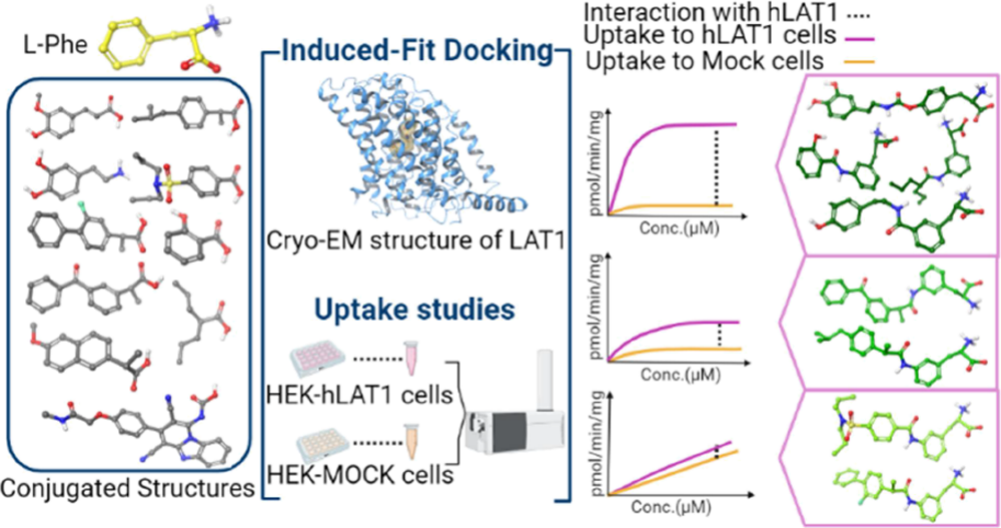

L-type amino acid transporter 1 (LAT1) transfers essential amino acids across cell membranes. Owing to its predominant expression in the blood–brain barrier and tumor cells, LAT1 has been exploited for drug delivery and targeting to the central nervous system (CNS) and various cancers. Although the interactions of amino acids and their mimicking compounds with LAT1 have been extensively investigated, the specific structural features for an optimal drug scaffold have not yet been determined. Here, we evaluated a series of LAT1-targeted drug-phenylalanine conjugates (ligands) by determining their uptake rates by in vitro studies and investigating their interaction with LAT1 via induced-fit docking. Combining the experimental and computational data, we concluded that although LAT1 can accommodate various types of structures, smaller compounds are preferred. As the ligand size increased, its flexibility became more crucial in determining the compound’s transportability and interactions. Compounds with linear or planar structures exhibited reduced uptake; those with rigid lipophilic structures lacked interactions and likely utilized other transport mechanisms for cellular entry. Introducing polar groups between aromatic structures enhanced interactions. Interestingly, compounds with a carbamate bond in the aromatic ring’s para-position displayed very good transport efficiencies for the larger compounds. Compared to the ester bond, the corresponding amide bond had superior hydrogen bond acceptor properties and increased interactions. A reverse amide bond was less favorable than a direct amide bond for interactions with LAT1. The present information can be applied broadly to design appropriate CNS or antineoplastic drug candidates with a prodrug strategy and to discover novel LAT1 inhibitors used either as direct or adjuvant cancer therapy.

## Introduction

The specific expression pattern of transporter families throughout the body represents a powerful strategy for drug delivery. By developing drug conjugates that could selectively bind to a specific transporter, the drug’s pharmacokinetic properties can be altered. Such an approach can ultimately slow down the progress of some diseases.^1−3^ This explains why transporter-mediated drug delivery has recently received increasing attention. In this respect, the large neutral amino acid transporter 1 (LAT1), due to its specific expression pattern under both normal and pathological conditions, holds great promise in the treatment of various types of illnesses.^4−6^

LAT1 (SLC7A5) is a sodium-independent amino acid exchanger.^7^ It forms a complex with a larger surface glycoprotein, 4F2hc (SLC3A2), to transport several neutral, branched L-type amino acids such as phenylalanine, tyrosine, and leucine.^5^ In healthy individuals, LAT1 is expressed as much as 100-fold greater levels in the blood–brain barrier compared to other tissues, providing a novel strategy for delivering amino acid-mimicking drugs to the central nervous system (CNS).^8^ Furthermore, LAT1 is expressed in the parenchymal brain cells, which subsequently enables intrabrain drug delivery.^5,9^ Notably, several drugs on the market reach the brain via this transporter; examples are gabapentin, L-DOPA, and baclofen.^10^

Furthermore, upregulation of LAT1 has been detected in primary tumors and metastatic lesions of various tumor origins.^11^ This finding means that LAT1 is an intriguing target also for cancer treatment. Inhibiting LAT1 can pave a way for suppressing tumor growth; this approach can also be exploited for the delivery of antineoplastic drugs into cancer cells via a prodrug strategy.^5,9^ Regarding the overexpression pattern of LAT1 in various cancers, developing radiolabeled selective LAT1 ligands could be one way to diagnose the presence of malignant cells in peripheral tissues.^12,13^

Despite the wide range of LAT1 applications with high expectations for drug delivery to the CNS and in cancer therapy, there are still gaps in our knowledge of the structural features which distinguish a LAT1 substrate from an inhibitor.^14^ Our group has broad experience in LAT1-targeted drug discovery, and our previous studies have led to the development of the three-dimensional (3D) pharmacophore^15^ and quantitative structure–activity relationship models.^16^ These studies have highlighted some structural features responsible for efficient LAT1 binding. To date, we and others have shown that recognition by this transporter requires the presence of free carboxyl and amine groups.^17,18^ Additionally, meta-substituted derivatives of phenylalanine exhibited a higher affinity for LAT1 compared to *ortho*- and para-substituted compounds.^19^

This study investigated a series of recently designed and synthesized phenylalanine drug conjugates. We have applied an integrated approach consisting of experimental *cis*-inhibition and uptake studies with a wild-type MCF-7 human breast cancer cell line, state-of-the-art Flp-In-HEK293-hLAT1/4F2hc-transfected cell line (hereafter HEK-hLAT1), and computational induced-fit docking (IFD) based on the recent cryo-electron microscopy (cryo-EM) structure of LAT1.^20^ Thus, we aim to understand how the electronic and spatial characteristics of a drug conjugated to phenylalanine will affect the compound’s interactions with LAT1. We also investigated this property with different bonds at various positions of the phenyl ring, which will lead to the elucidation of the structure–function relationship.

The present information clarifies how the linker structure as well as the distribution of polar and lipophilic regions of the conjugated drug scaffold affects a compound’s interactions and uptake via LAT1. This study also sheds light on specific features that medicinal chemists should consider when designing LAT1-targeted drug conjugates.

## Experimental Section

### Establishment of the Stably LAT1/4F2hc Expressing Cell Line

HEK293 Flp-In host cells were obtained from Invitrogen and maintained in Dulbecco’s modified Eagle’s medium (DMEM; Gibco, ThermoFisher Scientific, Waltham, MA, USA) supplemented with l-glutamine (2 mM; ThermoFisher Scientific, Waltham, MA, USA), heat-inactivated fetal bovine serum (10%, Gibco, ThermoFisher Scientific, Waltham, MA, USA), and penicillin (50 U/mL)-streptomycin (50 μg/mL) solution (ThermoFisher Scientific, Waltham, MA, USA), under humidity with 5% CO_2_ at 37 °C.

The pcDNA5-LAT1-4F2hc-FRT plasmid was constructed in GeneCopoeia/LabOmics (LabOmics S.A. Rue du Progrès, 4, BE-1400 Nivelles, Belgium). The human cDNA ORF clone sequences for LAT1 (SLC7A5) and 4F2hc (SLC3A2) were inserted in the pcDNA5/FRT mammalian expression vector. LAT1 and 4F2hc cDNAs are linked together with an internal ribosome entry site which enables transcription and translation of both proteins from the same plasmid. This plasmid is designed for the generation of stable cell lines by specifically using Flp-In host cell lines. HEK293 Flp-In cells were seeded onto 100 mm dishes at a density of 1 × 10^6^ cells/dish and cultured for 48 h. Thereafter, cells were transfected by using Lipofectamine 2000 LTX (ThermoFisher Scientific, Waltham, MA, USA) according to the manufacturer’s instructions. One day after transfection, fresh medium supplemented with hygromycin B (100 μg/mL) was changed. The cells were cultured in a medium supplemented with hygromycin B (medium change every 3–4 days) until single-cell colonies were observed in 2–3 weeks. Single colonies were collected using 0.25% trypsin-EDTA, transferred to 96-well plates, and cultured in the hygromycin B-containing selection medium. Confluent cells were then transferred to 24-well plates, further to six-well plates, and finally to 100 mm dishes. Cells stably expressing human LAT1 were then tested with [^14^C]-L-leucine, and the subclone showing the highest [^14^C]-L-leucine transport activity was selected for further analysis (Supporting information, Figure S2).

### Cell Culture

Cells were cultured in DMEM (Gibco, ThermoFisher Scientific, Waltham, MA, USA) supplemented with l-glutamine (2 mM; ThermoFisher Scientific, Waltham, MA, USA), heat-inactivated fetal bovine serum (10%; Gibco, ThermoFisher Scientific, Waltham, MA, USA), penicillin (50 U/mL)-streptomycin (50 μg/mL) solution (ThermoFisher Scientific, Waltham, MA, USA), and hygromycin B solution (100 μg/mL) for transfected cells. HEK-hLAT1 and HEK-MOCK cells (passages 7–20) were seeded at a density of 5 × 10^5^ cells/wells onto 24-well plates. The cells were used in the characterization, competition, and uptake studies 2 days after seeding. MCF-7 cells were seeded at a density of 1 × 10^5^ and were used in the competition studies 1 day after seeding. The culture medium was removed, and the cells were washed with prewarmed Hanks’ balanced salt solution (HBSS; 125 mM NaCl, 4.8 mM KCl, 1.2 mM MgSO_4_, 1.2 mM KH_2_PO_4_, 1.3 mM CaCl_2_, 5.6 mM d-glucose, and 25 mM HEPES, pH 7.4). The cells were then incubated with HBSS at 37 °C for 10 min before the experiments.

### Characterization of the Transfected Cell Line by Its LAT1 Function

HEK-hLAT1 cells were characterized by their LAT1 function with the known LAT1 substrate [^14^C]-L-leucine under different conditions. First, the time-dependent uptake was determined to define the optimal incubation time for the uptake of [^14^C]-L-leucine. HEK-hLAT1 cells (passages 7–20) were seeded at a density of 5 × 10^5^ cells/wells onto 24-well plates. The cells were used in characterization studies 2 days after seeding. The LAT1 characterization for the MCF-7 cells is described in refs (21, 22).

The cells were washed with prewarmed HBSS, and the cells were preincubated for 10 min before the experiments. To study the time-dependent uptake of [^14^C]-L-leucine, the cells were then incubated for 10 different time points (0.5–60 min, *n* = 4) with 250 μL of HBSS including 0.76 μM (0.1 mCi/mL) [^14^C]-L-leucine. After the incubation, the reaction was stopped with 500 μL of ice-cold HBSS, and the cells were washed twice with 500 μL of ice-cold HBSS. The cells were then lysed with 250 μL of 0.1 M NaOH for 60 min at RT. The lysate was mixed with 1.0 mL of Emulsifier safe cocktail (PerkinElmer, Waltham, MA, USA), and radioactivity was measured by a liquid scintillation counter (MicroBeta^2^ counter, PerkinElmer, Waltham, MA, USA). The optimal incubation time was determined from the linear range of the time-dependent uptake curve with a 10-min incubation time being selected for studies on dependency on the substrate and Na^+^-free concentrations, pH, and temperature.

The protocol for the cell culture and the uptake experiments was the same as described above with the exception of some changes in incubation buffers and conditions. For the concentration-dependent characterization, [^14^C]-L-leucine was used at concentrations of 0.76–500 μM in the HBSS buffer described above. Uptake was studied also in an ice bath at +4 °C, using the same concentrations (0.76–500 μM) mixed with ice-cold HBSS buffer. Leucine uptake was then determined in Na^+^-free buffer by replacing NaCl with equimolar choline chloride (125 mM). Finally, the uptake was studied also under different pH conditions (pH 4.5; 5.5; 6.5; 7.4; and 8.5). HEPES was replaced with MES (2-(N-morpholino) ethanesulfonic acid) with an acidic pH.

To demonstrate the difference in LAT1 expression levels in HEK-MOCK and HEK-hLAT1 cell lines, the localization of the LAT1/4F2hc complex was confirmed with immunofluorescence staining as well as quantifying the content of LAT1 protein. In immunofluorescence staining, the cells were cultured in Ibidi μ-Slide eight wells (IBIDI Gmbh, Germany) at a density of 50,000 cells/well. Two days after seeding, the cells were fixed with 150 μL of 100% MeOH at −20 °C overnight. The cells were stained on the next day after fixing. Shortly, the cells were washed with 0.1% bovine serum albumin (BSA), blocked, and permeabilized with 300 μL of 1% BSA with 0.1% Triton X-100 at RT for 45 min. The cells were incubated with primary antibody dilutions 1:50 at RT for 1.5 h (LAT1#5347 for LAT1, Cell Signaling and AM33318PU-T for 4F2hc, Origene). After primary antibody incubation, the cells were washed twice with 300 μL of 0.1% BSA, secondary antibody was prepared (Alexa Fluor 594 for LAT1, Alexa Fluor 488 for 4F2hc), and the cells were incubated at RT for 1 h in the dark. After the incubations, the cells were washed with 1xPBS and imaged with a Zeiss LSM 800 Airyscan confocal microscope (Carl Zeiss Microimaging GmbH, Jena, Germany).

To quantify the LAT1 protein amount from crude membrane fractions of HEK-MOCK and HEK-LAT1 cells, the sample preparation was performed as described previously.^23,24^ Shortly, the Membrane Protein Extraction Kit (BioVision Incorporated, Milpitas, CA, USA) was used for the extraction of crude membrane fractions from the cell pellet according to the manufacturer’s protocol. The protein concentration was measured using a Bio-Rad Protein Assay (EnVision, PerkinElmer, Inc., Waltham, MA, USA), and 50 μg of protein from each sample (*n* = 3) was taken for further analysis. Further sample preparation was performed as described earlier,^23^ and the absolute LAT1 quantity was determined by an LC–MS/MS-SRM setup. In more detail, the quantification of LAT1 and the membrane marker Na^+^/K^+^ ATPase was based on three selected reaction monitoring (SRM) transitions of the precursor and product ions from both the light and heavy peptide chains (Supporting Information, Figures S4–S9), as previously described. A total of 20 μL of the digested peptides (10 μg) was injected into an Agilent 1290 LC system coupled with an Agilent 6495 triple quadrupole mass spectrometer with an electrospray ionization source operated in the positive mode (Agilent Technologies, Santa Clara, CA, USA). Initially, the peptides were separated on a 2.1 × 250 mm, 2.7 μm column (Agilent Technologies, Santa Clara, CA, USA) and eluted by a gradient of 0.1% formic acid in water (A) and acetonitrile (B). A constant flow rate of 0.3 mL/min was utilized, and the gradient was shifted in the following way: 2–7% B for 2 min, followed by 7–30% B for 48 min, 30–45% B for 3 min, and 45–80% B for 2.5 min before re-equilibrating the column for 4.5 min. The data were acquired using the Agilent MassHunter Workstation Acquisition and processed using the Skyline software 20.1. The LAT1 and Na^+^/K^+^-ATPase proteins were quantified based on the ratio between the light and heavy peptides.

Finally, the uptakes of known LAT1 substrates L-DOPA, L-tryptophan, melphalan, gabapentin, and an inhibitor BCH were studied. The concentrations for the compounds were in the range of 2–400 μM. Otherwise, the experiment was carried out as described above and analyzed by liquid chromatography coupled with an electrospray ionization (ESI) triple quadrupole mass spectrometer (LC–MS–MS).

### Ability of Compounds To Inhibit LAT1

In the following experiments, the MCF-7, HEK-hLAT1, and HEK-MOCK cells were cultured, seeded, and preincubated as described above. The HBSS was removed, and the ability of the compounds to inhibit the uptake of a known LAT1 substrate, [^14^C]-L-leucine (PerkinElmer, Waltham, MA, USA), was studied by incubating the cells at RT for 10 min in uptake buffer pH 7.4 (250 μL) containing 0.76 μM (0.1 mCi/mL) of [^14^C]-L-leucine and 0.025–1800 μM of the studied compound (or HBSS as blank). After incubation, the reaction was stopped with ice-cold HBSS, and the cells were washed two times with ice-cold HBSS. The cells were then lysed with 250 μL of 0.1 M sodium hydroxide, the lysate was mixed with 1.0 mL of Emulsifier safe cocktail (PerkinElmer, Waltham, MA, USA), and the radioactivity was measured with a liquid scintillation counter (MicroBeta^2^ counter, PerkinElmer, Waltham, MA, USA). The inhibition of [^14^C]-L-leucine in the presence of the studied compounds as compared to the control (HBSS) was calculated as percentages (%).

### Time-Dependent Uptake Studies

Cellular uptake of the studied compounds into hLAT1-HEK and HEK-MOCK cells was determined at 100 μM concentrations at several time points (0.5, 1, 2, 5, 10, 15, 20, 30, 40, 50, and 60 min). The optimal incubation time was determined from the linear range of the time-dependent uptake curve, and the 10-min incubation was selected for further uptake studies.

### Concentration-Dependent Uptake Studies for hLAT1 and MOCK Cells

The concentration-dependent uptake studies were performed by adding 2–400 μM of the tested compound in 250 μL of prewarmed HBSS buffer on the cell layer. Incubation times for each compound were 10 min as determined in the time-dependent experiment. After incubation, the cells were washed and lysed as described above. The lysate was collected from wells into an Eppendorf tube and centrifuged at 4 °C, the supernatant was collected, and the intracellular concentrations of studied compounds were analyzed by liquid chromatography coupled with an ESI triple quadrupole mass spectrometer (LC–MS–MS). The results were calculated from the standard curve that was prepared by spiking known amounts of each compound to the cell lysate and normalized to protein concentration. The protein concentrations on each plate were determined as a mean of three samples by Bio-Rad Protein Assay, based on the Bradford dye-binding method, using BSA as a standard protein and measuring the absorbance (595 nm) by a multiplate reader (EnVision, PerkinElmer, Inc., Waltham, MA, USA). All statistical analyses (Michaelis–Menten kinetic parameters) were performed using GraphPad Prism v. 5.03 software (GraphPad Software, San Diego, CA., USA).

### Competition and Uptake Studies

Using the stably hLAT1/4F2hc expressing cell line, the compounds’ inhibitory efficiencies and uptake kinetics were determined via the *cis*-inhibition assay and uptake studies, respectively. (Table 1) *Cis*-inhibition is based on the competition between the ligand and LAT1 substrate [^14^C]-L-leucine that results in inhibition of substrate uptake. The competitive uptake with L-leucine is reported as the IC_50_ value of L-leucine uptake and extent as inhibition percentage (%) at a given concentration. Overall, the inhibition trends of the tested compounds were similar between HEK-hLAT1 and MCF-7 cells. In the next step, more straightforward results were obtained by undertaking direct cellular uptake studies with both HEK-hLAT1 and HEK-MOCK cells. This experiment enabled us to determine not only the uptake kinetics (Vmax and Km) of the ligands but also their preference for LAT1-expressing cells over the comparator HEK-MOCK cells.

**Table 1:**
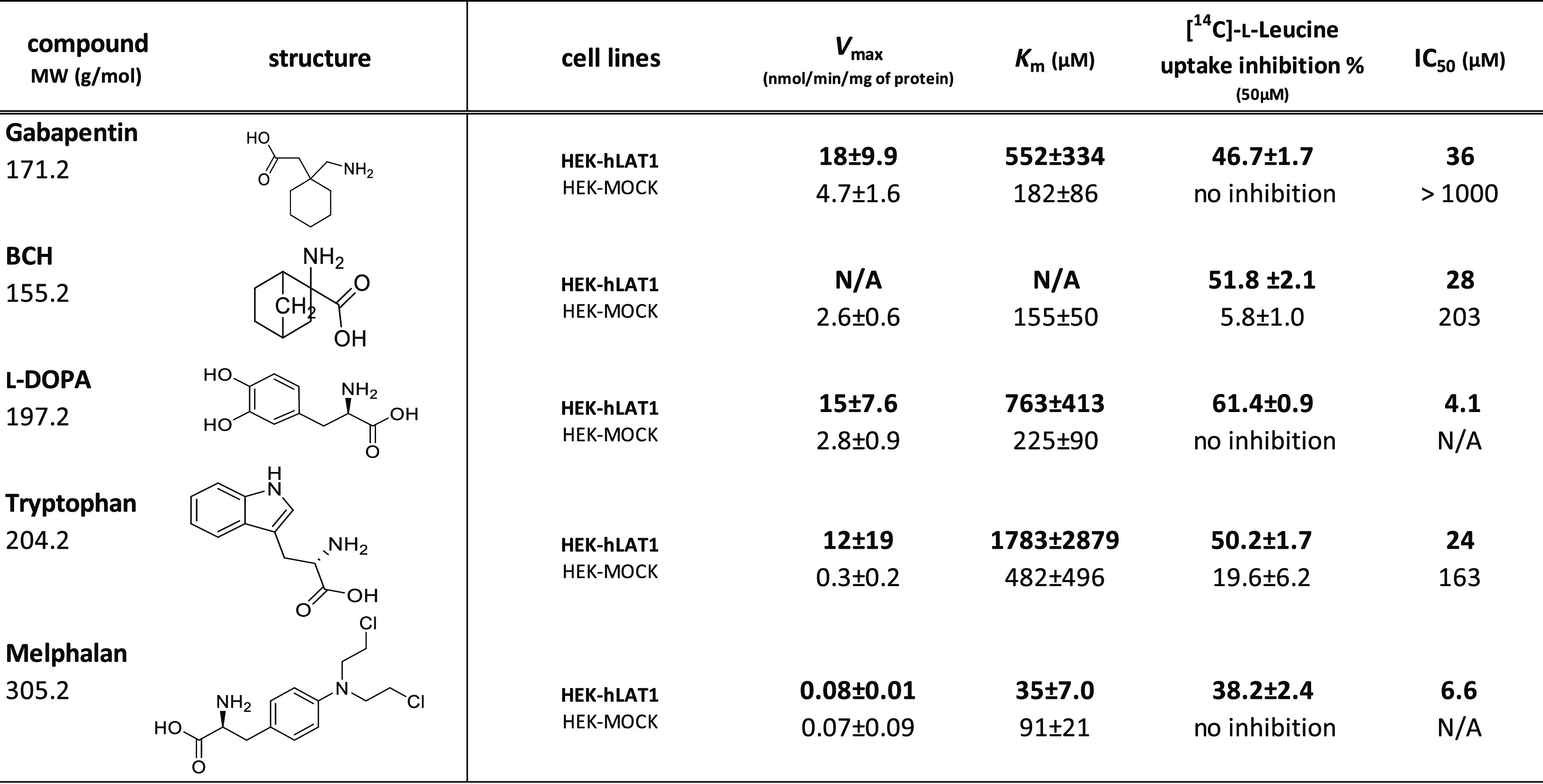
Calculated Inhibition Potency (IC_50_ & Inhibition%) and Uptake (Michaelis–Menten Enzyme Kinetics; *V*_max_ and *K*_m_) Values for Known LAT1-Substrates (*n* = 3)^a^

a HEK-hLAT1 is shown in bold and HEK-MOCK cells are in normal font.

### Sample Preparation and LC–MS/MS Analysis

For known LAT1 substrates (L-DOPA, L-tryptophan, melphalan, gabapentin, and inhibitor BCH) as well as for studied compounds **1–12**, sample preparation and analysis were carried out like described previously.^19,25−34^ Shortly, for L-tryptophan, melphalan, and compounds **2** and **4–12**, cells were lysed with 0.1 M NaOH, and an aliquot was diluted with acidified acetonitrile (0.1% formic acid) 1:4 including the internal standard (IS) diclofenac. After protein precipitation, the samples were centrifuged for 10 min with 10,000 g at 4 °C. The supernatant was then collected and transferred to vials for LC–MS/MS analysis. For BCH and gabapentin, cells were also lysed with 0.1 M NaOH, an aliquot was taken from the lysates, and perchloric acid (PCA) was added to precipitate the proteins. The samples were then centrifuged, and the supernatant was diluted 1:10 with H_2_0 with 0.1% formic acid including the IS and transferred to vials for analysis. For L-DOPA and compounds **1** and **3**, cells were lysed with 1% PCA, and the samples were centrifuged and diluted 1:4 with H_2_0 with 0.1% formic acid including the IS for analysis.

The samples were analyzed with LC–MS/MS coupled with an Agilent 1200 series Rapid Resolution LC System (Agilent Technologies, Waldbronn, Germany) and an Agilent 6410 Triple Quadrupole with electrospray ionization (ESI) (Agilent Technologies, Palo Alto, CA, USA). The samples were injected (1 μL for L-tryptophan, 5 μL for melphalan, BCH, gabapentin, L-DOPA, and compounds **1**–**12**) to the reversed-phase HPLC column (for tryptophan and melphalan: Zorbax Eclipse SB-C18 Rapid Resolution HT 2.1 × 50 mm, 1.8 μm, Agilent Technologies, Palo Alto, CA, USA, and for BCH, gabapentin and L-DOPA: Zorbax Eclipse XDB-C18 Rapid Resolution HD 2.1 × 100 mm, 1.8 μm, Agilent Technologies, Palo Alto, CA, USA, Zorbax Eclipse XDB-C18 Rapid Resolution 4.6 × 50 mm, 1.8 μm). The aqueous mobile phase was 0.1% formic acid in water (A), while the organic mobile phase was 0.1% formic acid in acetonitrile (B). The column temperature was 40 °C and the flow rate of 0.2 mL/min (for BCH, gabapentin, L-DOPA, and compound **8**), 0.3 mL/min (for melphalan, L-tryptophan and compounds **3** and **4**), 0.4 mL/min (for dopa-CBT), and 0.5 mL/min (for compounds **1–2**, **5–7**, and **9–12**) was used. The following gradients were used: for L-DOPA, L-tryptophan, and diclofenac 0–4 min: 2% → 35% B, 4–5 min: 35% → 95% B, 5–6 min 95%, 6–6.1 min: 95% → 2% B, 6.1–10 min: 2% B, for BCH, gabapentin and diclofenac 0–4 min: 5% → 90% B, 4–6 min: 90% B, 6–6.1 min: 90% → 5% B, 6.1–10 min 5% B, and for melphalan and diclofenac 0–4 min: 10% → 90% B, 4–6 min: 90% B, 6–6.1 min: 90% → 10% B, 6.1–10 min: 10% B, for compounds **1**, **7,** and **10–12**: 0-2 min: 5% → 95% B, 2–7 min 95%, 7–7.1 min 95% → 5% B, 7.10–10 min 5%, for compounds **2** and **3**: 0–3 min 5% → 60%, 3–3.5 min 60% → 95%, 3.5–6 min 95%, 6–6.1 min 95% → 5%, 6.1–9 min 5%. For compound **4**: 0–4 min 10% → 90% B, 4–6 min 90% B, 6–6.1 min 90% → 10%, 6.1–9 min 10%. For compound **5**: 0–1 min 20% → 95% B, 1–4 min 95%, 4–4.1 min 95% → 20%, 4.1–7 min 20%. For compounds **6** and **9**: 0–2 min 10% → 95%, 2–6 min 95%, 6–6.1 min 95% → 10%, 6.1–9 min 10%. For compound **8**: 0–1 min 20% → 80%, 1–5.2 min 80%, 5.2–5.3 min 80% → 20%, 5.3–9 min 20%. The following instrument optimizations were used: 300 °C sheath gas heat, 6.5 L/min drying gas flow, 25 psi nebulizer pressure, and 3500 or 4000 V (for compounds **1**, **5**, **7–8**, and **10–12**) capillary voltage, respectively. Detection was performed by using multiple reaction monitoring with the following transitions: *m/z* 205.1 → 146.1 for L-tryptophan, *m/z* 198.1 → 152 for L-DOPA, *m/z* 156 → 110 for BCH, *m/z* 172 → 137 for gabapentin, *m/z* 305 → 288 for melphalan, and *m/z* 296.1 → 250 for diclofenac.

The lower limit of quantification (LLOQ) for gabapentin and L-tryptophan was 2.5 and 5.0 nM for L-DOPA, BCH, and melphalan, respectively.^29^ These LC–MS/MS methods were also selective, accurate (RSD < 15%), and precise (RSD < 15%) over the range of 5.0–2000 nM for melphalan; 5.0–6000 nM for BCH; 2.5–6000 nM for gabapentin; and 5.0–10,000 nM for L-DOPA and L-tryptophan. For compound **9**, the LLOQ was 0.05 nM with good linearity (*R*^2^ > 0.983).^34^ For the compounds **1**, **7**, and **12**, the LLOQ was 12.5 nM, with a linear range of 12.5–2500 nM, and for **11**, the LLOQ was 6.3 nM, over the range of 6.3–6250 nM.^28^ For **10**, the calibration curve was linear with a LLOQ of 1 ng/mL (accuracy ±15%), respectively.^26^ For compounds **2–5**, the LLOQ was 5.0 nM.^29^ Methods were also selective, accurate (RSD < 15%), and precise (RSD < 15%) over the range of 5.0–1000 nM for **5** and for compounds **2–4** 5.0–2000 nM. For compound **6**, the LLOQ was 10 pmol/g.^33^ Ketoprofen was linear with a range of 0.25–25 ng/mL, while compound **8** was completely converted to ketoprofen in 0.1 M NaOH within 30 min.

### Protein and Ligand Preparation for IFD

Molecular docking was performed using the Schrödinger maestro suite 2021-04 (Schrodinger release 2021-4: Protein preparation wizard, Schrodinger, LLC, New York, NY, 2021). The electron microscopy structure of LAT1 (PDB ID: 7DSQ)^20^ was prepared by the protein preparation wizard. Bond orders were assigned, hydrogens were added, het states were left in default PH (7.0 ± 2.0) using Epik, and all other settings were kept as default. Thus, the initial docking was carried out using SP (standard precision) settings, OPLS4 force field, and the default 0.5 scaling for van der Waals interactions for both ligands and receptors. The prime module was further used to account for protein flexibility by refining protein conformations within 5 Å from initial docking poses. Glide redocking was finally computed using the SP level of settings resulting up to 20 poses. Except for **9** and **10** for which we used the constraint to oxygen of carboxyl group of Gly255 and hydrogen of amino group of Gly67, no constraint was used for the rest. Also, because of the bigger size of **6,** the dimensions of the grid box were increased to 20 Å for this compound. The ligand preparation was accomplished using the LigPrep OPLS4 force field. Possible states were generated at pH (7.0 ± 2.0) using Epik, and ligands with the *S*-enantiomer of phenylalanine were minimized by the ligand minimization module (Schrodinger release 2021-4: Protein preparation wizard, Schrodinger, LLC, New York, NY, 2021).

## Results

### Development of the Stably hLAT1/4F2hc Expressing Cell Line

The subclone with the highest [^14^C]-L-leucine transport capacity was selected and further characterized. The functionality and the transport properties of HEK-hLAT1 cells, as compared to HEK-MOCK cells, were demonstrated in in vitro *cis*-inhibition and uptake experiments with known LAT1 substrates such as L-DOPA, BCH, melphalan, gabapentin, and L-tryptophan (Table 1, Figure S1). All the studied compounds were able to compete with [^14^C]-L-leucine in the *cis*-inhibition study and showed higher binding interactions with HEK-hLAT1 cells (lower IC_50_ values and higher inhibition%) than in HEK-MOCK cells (Table 1). These IC_50_ values were also lower than those reported in previous studies with MCF-7 cells.^22,31,33^ Analogously, the compounds were taken up much more effectively into HEK-hLAT1 cells compared to HEK-MOCK cells. However, because of the different end point readings of inhibition and uptake experiments some extent of data discrepancy might be observed, and data interpretation should be done cautiously.

**Figure 1:**
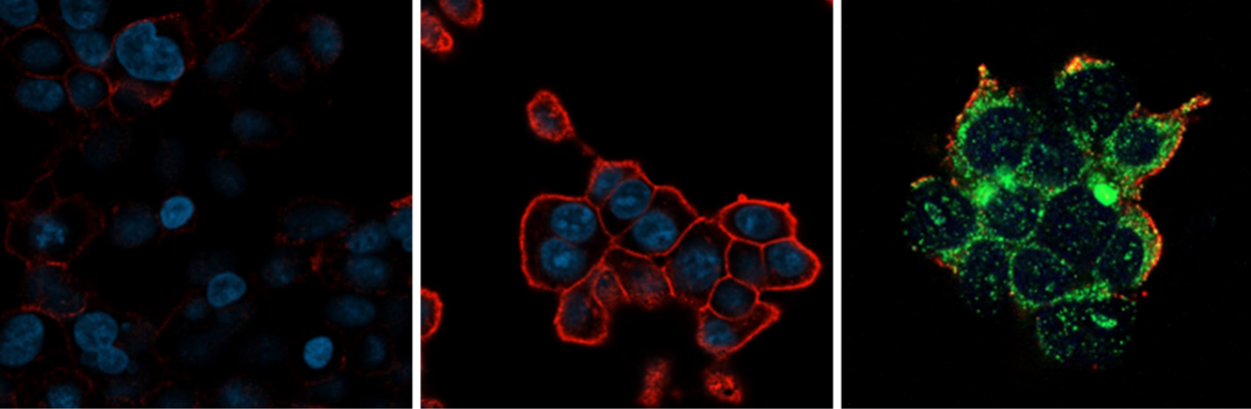
Confocal microscopy images after immunofluorescence staining of HEK-MOCK cells (left) and HEK-hLAT1 cells (middle and right), showing LAT1 localization in the cell membrane (LAT1 = red, nuclei of the cells = blue, 4F2hc = green, overlapping of LAT1/4F2hc = yellow/orange).

The amount of transporter protein was quantified by LC–MS/MS-SRM from both cell lines in order to confirm the expression levels of the LAT1-4F2hc heterodimeric complex. The content of LAT1 in HEK-hLAT1 cells was 0.38 ± 0.06 fmol/μg protein whereas LAT1 was not detectable in HEK-MOCK cells. In addition, the content for 4F2hc in HEK-hLAT1 cells was 0.49 ± 0.07 fmol/μg protein, much higher than in HEK-MOCK cells (0.02 ± 0.001 fmol/μg of protein) (Figures S4–S9). The expression levels for LAT1 and 4F2hc were at similar levels in human breast cancer (MCF-7) cells compared to HEK-hLAT1 cells.^5^ In addition, immunofluorescence staining demonstrated the large differences in LAT1 expression between HEK-MOCK and HEK-hLAT1 cell lines (Figure 1). In summary, LAT1 is highly expressed in HEK-hLAT1 cells, while its expression in HEK-MOCK cells is negligible. To determine the functional difference between these two cell lines, we also measured the amount of [^14^C]-L-leucine uptake under different conditions (Figure S2). It is worth considering that L-leucine may also use other transport mechanisms like LAT2 (SLC7A8)^35,36^ or B0AT2 (SBAT1, SLC6A15),^37^ and it is one of the limitations of our study that the substrate is not 100% selective for LAT1. However, concentration-dependent uptake by L-leucine is the best choice we found so far for this purpose.

### Compound Selection

Our group has so far developed several drug conjugates capable of LAT1-mediated drug delivery. Most of the compounds described in this study were phenylalanine meta-substituted conjugates. The detailed synthesis descriptions have been reported elsewhere (compounds **1**, **7**, **11** and **12,**(28)**2,**(31)**3,**(38)**4,**(19)**5,**(9)**6,**(33)**8,**(39)**9,**(34)**10**(40)). Therefore, the interactions of the selected compounds with the LAT1 transporter and their kinetic parameters (Km, Vmax) were determined by experimental assays and IFD as described below (Table 2).

**Table 2:**
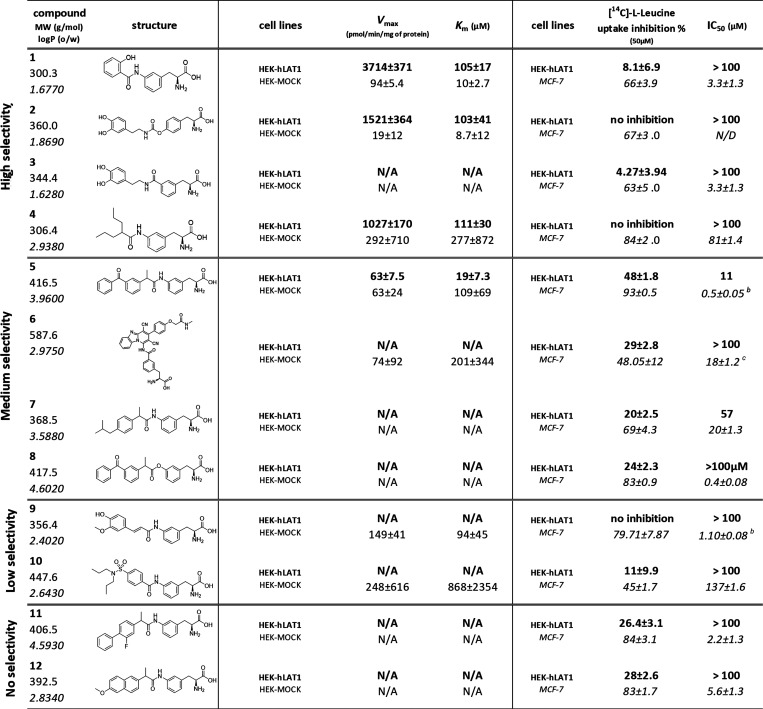
Results of Uptake Studies *V*_max_ (pmol/min/mg of Protein) and *K*_m_ and *cis*-Inhibition Assay with Different Cell Lines^a^

a The HEK-hLAT1 cell line is shown in bold, HEK-MOCK cells in normal font and MCF-7 cells in italics.

b IC_50_ values in MCF-7 cells published in ref (22).

c IC_50_ values in MCF-7 cells published in ref (33).

Based on the difference in their uptake into HEK-hLAT1 and HEK-MOCK cells, coupled with consideration of their conformation and protein interaction patterns in docking studies, the compounds could be classified into four groups: **1** (salicylic acid derivative), **2** and **3** (dopamine derivatives), and **4** (valproic acid derivative) showed the highest interactions; **5** and **7** (ketoprofen and ibuprofen conjugates, respectively) and **6** (LAT1 inhibitor KMH-233) displayed medium interactions, whereas the interactions of **9** and **10** (ferulic acid and probenecid derivatives, respectively) were low, and finally, compounds **11** and **12** (flurbiprofen and naproxen conjugates, respectively) were not interacting with LAT1. It is worth mentioning that the word interaction in this classification refers to both interactions of the compounds and their transportability via LAT1.

#### Compounds with High Interactions

The highest uptake rate and interactions in this group were observed with compound **1**, while **2** and **4** were ranked next, and the lowest uptake and interactions were evident with **3**.

**Figure 2:**
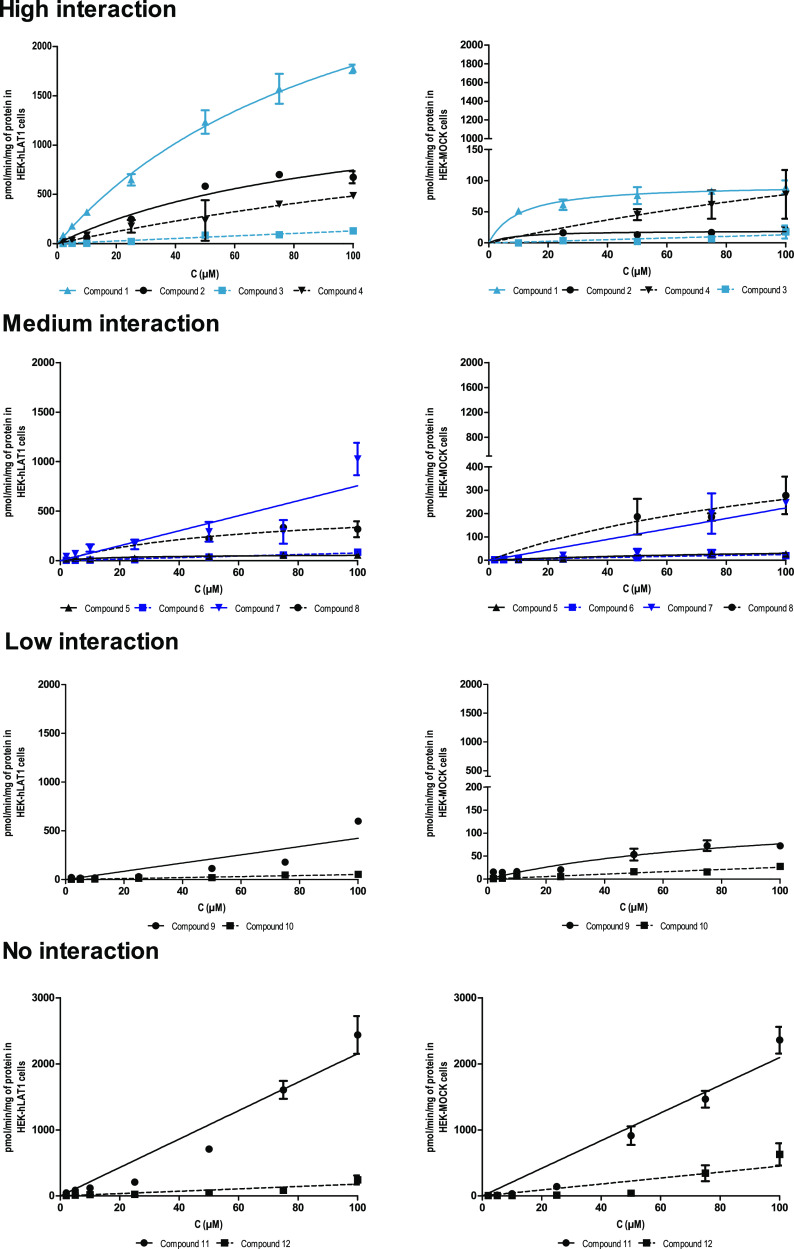
Uptake results of compounds (**1**–**12**). HEK-hLAT1 cells presented with circles (solid black line, values on the left *Y* axis), HEK-MOCK cells with squares (blue dashed line, values on the right *Y* axis). The *Y* axis is adjusted according to each compound.

The good interactions of the first group of compounds with various structures reflected the high tolerance of LAT1 for transporting different hydrophobic and hydrophilic moieties. The higher interactions and uptake rate of **1** over **2** and **3** with the catechol hydroxyl group could be attributable to its smaller size (Figure 2).

Notably, there was a significant difference between the uptake and interactions of dopamine conjugates **2** and **3**. Because Michaelis–Menten saturation was not achieved at concentrations studied (<100 μM), Vmax and Km values could not be reported for **3** in which dopamine is attached at the meta-position of phenylalanine by a reverse amide bond. In contrast, compound **2**, where dopamine is attached at the para-position of the phenyl ring by a carbamate bond, displayed a high Vmax, and its Km value was very similar to that of **1**. The uptake rate of **2** into HEK-hLAT1 cells (1521 pmol/min/mg) was 75 times greater than its uptake into HEK-MOCK cells (19 pmol/min/mg). Interestingly, in previous studies with MCF-7 cells, both of these compounds displayed a very low Vmax value (<3 pmol/min/mg), and at concentrations above 50 μM, it was assumed that some other transport mechanisms such as an organic anion transporter were involved in their transport.^14^ According to these previous studies, **2** was already assumed as a promising drug candidate for Parkinson’s disease because of its higher stability than L-DOPA in rat liver homogenate, and also the capability of its tyrosine promoiety to be converted into L-DOPA.^31^ More specific investigation in this study provides further proof for previous findings by others.

Compound **4**, which is a valproic acid derivative, also showed good interactions with hLAT1. Its Vmax in HEK-hLAT1 cells (1027 pmol/min/mg) was around three times higher than in HEK-MOCK cells (292 pmol/min/mg), and it displayed more than two-fold higher potency for hLAT1 (Km of 111 μM for HEK-hLAT1 vs 277 μM for HEK-MOCK cells).

Previous in situ rat brain uptake experiments demonstrated that amide derivatives of phenylalanine and valproic acid had higher uptakes than their corresponding ester derivatives.^32^ Furthermore, meta-substituted amide derivatives displayed a higher uptake than their para-substituted counterparts. It was also demonstrated that the addition of one methylene spacer between the aromatic ring and amide bond increased uptake into MCF-7 cells (48 vs 28 pmol/min/mg).^32^ Even though we did not analyze the effect of the methylene spacer on direct uptake in this study, we did observe a very good interaction and uptake profile with the valproic acid derivative **4**, which reflects the high capacity of LAT1 for transporting various types of structures.

#### Compounds with Moderate Interactions

Compounds **6** (LAT1 inhibitor) and **5** and **7** (amide conjugates of ketoprofen and ibuprofen, respectively) were ranked as a second group in terms of interactions with hLAT1 over HEK-MOCK cells. Compound **8**, an ester analogue of **5**, displayed virtually no interaction for hLAT1 at up to 50 μM concentrations although at higher concentrations, a secondary uptake mechanism was activated for this compound (Figure 2). It might be traced to other factors, such as a higher susceptibility of the ester conjugate, **8**, for hydrolysis or the utilization of other transport routes in our experimental setup given that it has a higher lipophilicity (*c*Log *P* 4.6) compared to **5**.

Compounds **5** and **7** displayed desirable interactions toward LAT1 with the overall uptake of **7** being higher. However, because its uptake to HEK-hLAT1 cells displayed a linear trend, kinetic parameters could not be calculated. Furthermore, a more than two-fold increase in its uptake rate was observed at and above 100 μM which implies participation of a secondary transport mechanism for **7**. In summary, compound **5**, despite its lower uptake rate, appeared to be a more selective LAT1 substrate. With respect to **7**, the absence of saturation and putative utilization of other transport mechanisms casts doubt on the hypothesis that it is a pure LAT1-utilizing compound (Figure 2).

The other compound in this group, **6**, a reversible LAT1 inhibitor designed and synthesized by our group, also showed moderate interaction toward LAT1.^33^ Its uptake into hLAT1 cells was 2 to 3 times higher than that in HEK-MOCK cells. This was especially evident in the concentration range of 50–100 μM, and its uptake became saturated at concentrations over 100 μM.

#### Compounds with Low Interactions

Compounds **9** and **10** (amide conjugates of ferulic acid and probenecid, respectively) exhibited only marginal interactions with hLAT1. Compound **9**, despite promising results in affinity studies with the MCF-7 cell line, was not able to inhibit L-leucine uptake in HEK-hLAT1 cells at a 50 μM concentration, and its IC_50_ value was also higher than 100 μM. Its interactions with hLAT1 were only slightly better than HEK-MOCK cells in the concentration range of 40–90 μM (Figure 2).

The other compound, **10**, was the most promising ligand among the probenecid conjugates; it had been previously studied in the MCF-7 cell line with a Vmax value of 111 pmol/min/mg and Km value of 15.16 μM.^40^ However, according to the *cis*-inhibition assay with MCF-7 and HEK-hLAT1 cell lines, **10** exhibited very low inhibitory potency (IC_50_ value >100 μM). The inhibition of L-leucine uptake at 50 μM concentration in these cell lines was 40 and 10%, respectively. In more specific uptake studies, this compound revealed no significant interactions for hLAT1 over HEK-MOCK cells within the concentration range of 2–100 μM rendering **10** unlikely to act as an effective LAT1-interacting compound (Figure 2).

#### Compounds with No Interactions

Compounds **11** and **12** (amide conjugates of flurbiprofen and naproxen) displayed the lowest interactions with hLAT1 in our experiments. Surprisingly, at concentrations above 60 μM, the uptake of **12** into HEK-MOCK cells even surpassed its uptake into hLAT1 cells (Figure 2).

The affinity of these two ligands toward MCF-7 cells was higher than that for hLAT1 cells in *cis*-inhibition assays. Because cancer cells highly express various transporters, this observation supports the idea that these compounds tend to utilize other transportation systems than LAT1 for their uptake. The lack of interactions of these two compounds could also be assigned to their high lipophilicity (log *P* 4.59) that enables them to gain access to the cells by different mechanisms, including passive diffusion. Previously, compound **11** had also revealed a remarkably high uptake with a linear trend in human immortalized microglia cells (SV40), primary mouse astrocytes, and BV2 cells.^28^ However, in the presence of a LAT1 inhibitor, the uptake of **11** was reduced at concentrations higher than 50 μM but was not affected at lower concentrations (<25 μM) which indicates that the LAT1 might only be involved in higher concentrations. In human SV40 cells, the uptake was even increased in the presence of the LAT1 inhibitor.^28^ This contradictory behavior may be due to the participation of other transport routes. The uptake of compound **12** was much lower than that of **11** and was not affected by the LAT1 inhibitor in the aforementioned cell lines.^28^ This finding together with minor interactions with HEK-MOCK cells we observed here rules out the possibility of **12** being a substrate for hLAT1.

### Induced-Fit Docking

The cryo-EM structure of LAT1 (Protein Data Bank ID:7DSQ) with outward-facing conformation was prepared and refined using the Protein Preparation wizard in Maestro Schrödinger and was used in the IFD studies with commercial compounds and phenylalanine conjugates in this study. According to the literature, Phe252 and Gly255 in transmembrane helix 6 (TM6) and a conserved Gly65-Ser66-Gly67 (GSG) residue motif in TM1 play an important role in ligand recognition and transport.^2^ It is worth mentioning that even though the docking score of the compounds ranged between −8 and −12 which are considered numerically favorable values in Maestro Schrödinger, we could barely find a clear relationship between the compounds’ behavior in vitro and their docking score. Regarding the limitations associated with the implementation of docking scores,^41^ favorable docking poses were selected based on the similarity of the ligands’ interaction pattern and binding mode to the cocrystallized ligand, Diiodo-tyrosine,^20^ and to that of specific, well-known LAT1 substrates (e.g., L-DOPA, gabapentin, phenylalanine, and tyrosine) with the current cryo-EM structure. Selected poses are publicly available and can be reached via (https://zenodo.org/record/7143457#.Yz0ftHZByUl).

**Figure 3:**
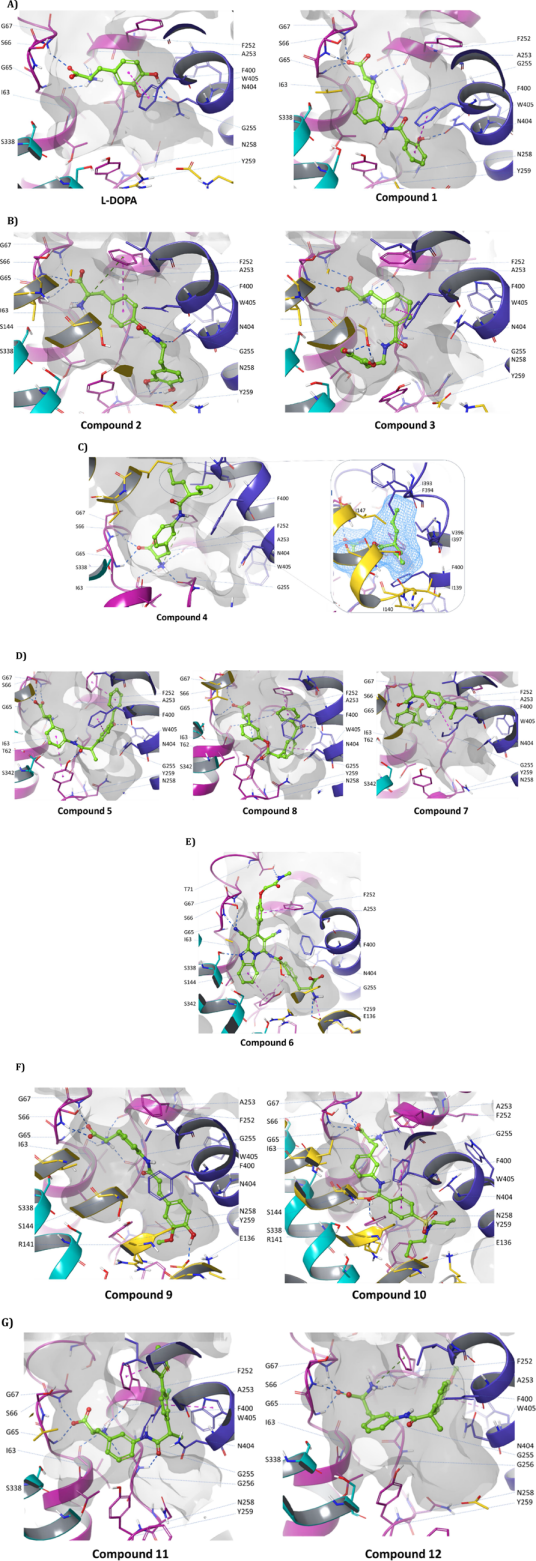
Favorable docking poses of compounds to the LAT1 cryo-EM structure. TM1 and TM6 are shown in pink, TM3, TM8, and TM10 are shown in yellow, cyan, and blue, respectively. (A) L-DOPA and compound **1**, (B) compounds **2** and **3**, (C) compound **4**, (D) compounds **5**, **8**, and **7**, (E) compound **6**, (F) compounds **9** and **10**, and (G) compounds **11** and **12**.

Within the first group, **1** displayed convergent docking poses. Its amino acid ending was well recognized by TM1 and 6. Similar to the well-known LAT1 substrates, L-DOPA, its phenol ring, and catechol hydroxyl were extended toward TM10 establishing interactions with Phe400 and Asn404 (Figure 3A).

Considering IFD results of dopamine conjugates, **2** and **3**, we observed that **2** was extended toward TM6 with the amine of its carbamate bond making H-bonds with Asn404 of TM10, and the ending catechol OH groups interacting with deeper residues of TM6 (Asn258). The other conjugate, **3**, with reversed amide bonds at the meta position was bent toward TM3 with its catechol OH groups interacting with Ser144 (Figure 3B).

Compound **4** despite having a different structure in this group still fits well inside the cavity. Its amino acid ending established H-bonds with the recognition site of the transporter, and its aliphatic chain occupied the adjacent cavity. It was surrounded by hydrophobic residues of the upper part of TM3 and TM10 (Ile139, Ile140, Ile147, Ile393, Phe394, Val396, Ile397, and Phe400) (Figure 3C).

Among the second group of compounds, we barely found convincing evidence by IFD studies for explaining the difference between amide and ester conjugates of ketoprofen, **5,** and **8**, respectively (Figure 3D). Although the amide bond of **5** has higher capability for making H-bonds than the ester of **8**, it may not be solely responsible for the higher interaction and potency (higher extent of inhibition and lower IC_50_ value in *cis*-inhibition assay) of **5** over **8**. It might root in other biological reasons like lower stability of ester conjugates as described in the previous section.

According to uptake studies, we concluded that **5** has a higher preference for LAT1 than **7**. Comparing binding mode of **5** with **7** in IFD studies, we speculate that the aromatic ring and the carbonyl group of **5** by providing this compound with more opportunity to interact with various aromatic and polar residues of LAT1 might contribute to its higher preference (Figure 3D). These structures in **5** are replaced with an isopropyl group in **7** which due to its smaller size can lead to a higher uptake rate of **7** whether by LAT1 or other transportation mechanisms.

Compound **6**, the reversible inhibitor of LAT1, shows moderate interactions in this study. Despite having a fairly big structure, it was able to fit inside the transportation path. While the larger moiety occupied the main transportation path, the phenylalanine moiety was accommodated in the cavity surrounded by polar residues of TM6, TM3, and TM10 establishing interactions with Glu136 and Tyr259 of TM3 and TM6 (Figure 3E). Simultaneous occupation of two regions leads **6** getting stuck like a hook inside the transporter and blocking the transportation path. Our observation in IFD studies was consistent with findings from previous in vitro studies which found it as a slowly reversible inhibitor of LAT1 that could be detached from the surface of the MCF-7 cells by warm wash.^31^ Furthermore, its overall binding mode and moderate interactions show how distribution of hydrogen bond acceptor (HBA) and hydrogen bond donor groups within a big structure can help gaining access to LAT1.

Compounds **9** and **10** have long and linear structures which led them to adopt a different binding mode and extend toward a deeper part of TM6, 3, and 10. Catechol OH of **9** established H-bonds with Glu136 of TM3, and the SO_2_ group of **10** did not make H-bonds with its surrounding residues. Also, its aliphatic tail was surrounded by hydrophilic residues of TM3 and 10 (Figure 3F).

Compounds **11** and **12** have a very similar 3D structure. The biphenyl structure of **11** occupied very similar spatial coordinates as the naphthalene ring of **12** to observed by ligand alignment (Supporting Information, Figure S12.a). Although they were recognized by their amino acid ending, it seems that reduced flexibility of the aromatic rings and lack of appropriate hydrophilic groups result in their reduced interactions in this study (Figure 3G).

## Discussion

In this study, we investigated both experimentally and computationally how the structural features of the drug will affect the drug-phenylalanine conjugate’s interactions with and transportability via LAT1. We observed that LAT1 can accommodate compounds with various aliphatic or aromatic moieties. However, smaller compounds still displayed the highest interactions as it was observed with **1**, which is the closest compound in size to the commercial ones, with one polar catechol OH group in its structure.

Compounds **1**, **2**, and **3** with a phenolic OH group displayed highest interactions. Similar to L-DOPA, the OH group of **1** established H-bond with Asn404 of TM10, corresponding structure in **2** made H-bond with a deeper part of TM6 (Asn258), and in **3** it was hydrogen bonded with Ser144 of TM3. Compound **9** also includes the phenolic hydroxyl group, but its specific 3D structure which is most probably due to the presence of the planar amide bond adjacent to the planar unsaturated ethylene group led this compound to adopt a different binding mode and caused its phenolic hydroxyl group to maintain H-bond with Glu136 in deeper part of TM3. Although **9** was also recognized well in this IFD study, it seems that its different more rigid conformation limits its interaction and transportability via LAT1. Interestingly, previous studies reported that the addition of one carbon atom spacer between the amide bond and the aromatic ring of the phenylalanine in **9** increased its uptake rate by more than 2-fold.^34^ This finding further emphasizes the importance of flexibility, especially for the transport of larger molecules.

Although **2** and **3** displayed better interactions and uptake characteristics than **9**, the superiority of **2** over **3** denotes how the chemical characteristics of the bond and the position of its linkage to phenylalanine affect the transport and interactions of the compound. One explanation for this observation might be that carbonyl group of the carbamate bond in **2** has higher HBA properties because it is located between two electron-donating atoms, oxygen and nitrogen whereas in **3** this carbonyl is directly attached to an electron-withdrawing phenyl group which reduces its HBA properties. It is also noteworthy that the phenolic hydroxyl of L-DOPA is replaced with electronegative nitrogen and oxygen atoms in **1** and **2**, while **3** possessed a carbon atom instead of it (Supporting Information, Figure S13). As a result, the distance between Cα and the carbon of the carbonyl group of the connecting bond for the first two compounds was around 7 Å. This matched with the currently approved LAT1 pharmacophore^15^ while this distance was reduced to 5 Å in **3**. In a recent study on ester conjugates of ketoprofen, direct positioning of the carbonyl group at the meta-position of phenylalanine was compared with the presence of a spacer oxygen group between the carbonyl and the phenyl ring in the para-position. It was observed that although the binding potency of the former was higher (lower IC_50_), the latter displayed greater substrate activity (i.e., higher efflux rate of *L*-phenylalanine in trans-stimulation assay).^42^ This finding, together with our observation, indicates that the presence of one spacer group at the para-position, especially within a carbamate bond, confers on the compound a better opportunity to interact with the transporter.

Compound **10** shares the same structural features with **4** in containing an aliphatic tail, but **10** displayed much lower interactions and uptake toward hLAT1. In compound **10**, the amide bond, the middle aromatic ring, and the sulfur atom of the sulfonamide group lie nearly in the same plane causing a kink and leading the aliphatic tail of **10** around 5 Å away from the corresponding structure in **4**. (Supporting Information, Figure S10) This specific arrangement of chemical groups dictates a distinct conformation which, similar to compound **9**, led the ending part of **10** to accommodate the cavity in deeper parts of TM3, TM6, and TM10. As a result, neither the aliphatic tail nor the SO_2_ group did not make appropriate hydrophobic interactions or H-bonds with their surrounding residues.

If we assumed a plane connecting the first and second aromatic features of **5**, **9**, and **10**, we could easily observe that there is around 78° of deviation between these two planes in **5**, while just a small deviation (0.6 and 5.1°) was observed for **9** and **10** (Supporting Information, Figure S11). This deviation helped the last two rings of **5** to maintain appropriate interactions especially with the gatekeeper residue Phe252 and might have contributed to its superiority over other two compounds. The lack of spacer carbon atoms between the amide bond and the second aromatic ring of **10** and the presence of a planar double bond between these two features in **9** formed the basis for their different 3D conformation which finally led to their different orientation inside the cavity and reduced interactions.

The lack of interactions of **11** and **12** was not solely related to their 3D conformation, and other factors like lipophilicity and nonspecific binding may also have contributed to this. However, when comparing their 3D structures and docking poses with that of **5** (Supporting Information, Figure S12.b), it can be inferred that both the position and orientation of the phenyl rings relative to each other and the presence of polar groups among them play an important role in determining the compounds capability to interact and inducing the conformational change of the transporter. In **11** and **12**, aromatic features lie in the same plane and have less freedom, whereas in **5**, the third phenyl ring is attached to the meta position of the second ring and occupies a completely different region. Undoubtedly, the flexibility and HBA properties of the carbonyl group attaching the two aromatic features of **5** also contribute to its higher interactions.

We also observed in this study that substituting the ending phenyl ring of **11** with an isobutyl group in **7** (Supporting Information, Figure S12.c) led to higher LAT1 interactions. This implies that the smaller size and greater flexibility of the isobutyl group had contributed to the higher hLAT1 interactions of **7**. Interestingly, both the affinity and uptake rate of **7** were lower than the corresponding values of **11** in previous studies conducted with human SV40 and mouse astrocytes as well as in BV2 cells.^14^ However, the lack of interactions of **11** in this study provided evidence that the high uptake observed here and in previous reports most probably indicated that **11** was utilizing other transporters and not necessarily LAT1.

An additional point to consider is that compounds **5**, **7**, **8**, **11**, and **12** in this study have two chiral centers that might also affect their biological behavior. It has been reported previously that LAT1 displays a higher affinity for the L- than for the D-form of amino acids.^43,44^ Although *L*-phenylalanine was utilized to synthesize the drug conjugates in this study, the chirality of the drug conjugate has not been completely clarified. The studied compounds were pure forms of either S, S, or S, R-enantiomers, but we did not test both enantiomers in parallel. Therefore, more studies are needed to determine which of the enantiomers is superior regarding LAT1-mediated transport.

In a nutshell, major findings of this study are as follows:

The presence of a double bond or other rigid structures adjacent to the planar amide bond reduced the interactions with LAT1. With the elongated compounds, a saturated ethyl group was preferred to unsaturated ethylene.

For compounds with more than one aromatic ring or planar group in their structure, the specific arrangement of these functional groups played a crucial role in determining their interactions. Attachment at the meta-position, especially if accompanied by specific polar spacer groups like carbonyl in **5**, confers the compound more flexibility and ability to interact with LAT1, whereas direct attachment of these two groups (like compounds **11** and **12**) prevents the compound from making the interactions necessary for transport.

Although previous studies suggest maintaining a distance of 3.8 Å between the HBA carbonyl group of the amide bond and the phenyl ring of the amino acid promoiety is in favor of LAT1 binding,^15^ we concluded that the overall structure of the compound will also affect compounds’ behavior. As an example, we observed that the interactions of **3** in which this distance was reduced as a consequence of the reverse amide bond were higher than that of **9** with the normal amide bond, but **9** has a planar double bond in the further part of its structure.

## Conclusions

With regard to the very broad application range of LAT1 in drug delivery to CNS and cancer cells, finding an appropriate drug scaffold for selective and efficient delivery via drug-phenylalanine conjugate strategy is of utmost importance. Our investigation addressed the lack of knowledge about the structural features of the drug scaffold and provided several clues on how to arrange different chemical groups to obtain high hLAT1 interactions and uptake. Considering the significance of selectivity in targeted drug delivery, these findings on how to increase interaction with LAT1 will assist all aspects of LAT1-targeted drug discovery from neurological diseases to the diagnosis and treatment of cancer.

## Supporting Information Available

More detailed information on uptake profiles of known LAT1 substrates, characterization of the novel transfected cell line by its LAT1 function, LAT1 protein expression levels, and ligand alignments (PDF)
