# Supplementary material for: Structural Features Affecting the Interactions and Transportability of LAT1-Targeted Phenylalanine Drug Conjugates
Source: Mol Pharm. 2022 Nov 17;20(1):206–18. doi: 10.1021/acs.molpharmaceut.2c00594 (PMC9811466; doi:10.1021/acs.molpharmaceut.2c00594)
Supplement: Supplementary file 1 — mp2c00594_si_001.pdf [file mp2c00594_si_001.pdf]

SUPPORTING INFO

Structural features affecting the interactions and transportability of LAT1-targeted phenylalanine drug conjugates

Katayun Bahrami[Ø*], Juulia Järvinen[Ø], Tuomo Laitinen, Mika Reinisalo, Paavo Honkakoski, Antti Poso, Kristiina M. Huttunen, Jarkko Rautio

AUTHOR ADDRESS. School of Pharmacy, University of Eastern Finland, P.O. Box 1627, FI-70211 Kuopio, Finland

[Ø]These authors contributed equally

TABLE OF CONTENTS

Uptake profiles of LAT1 substrates………………………………………………………………………….... 2

Characterization of LAT1……………………………………………………………………………………………. 3

LAT1 expression levels…………………………………………………………………………………………….. 4

Ligand alignment…………………………………………………………………………………………………….. 7

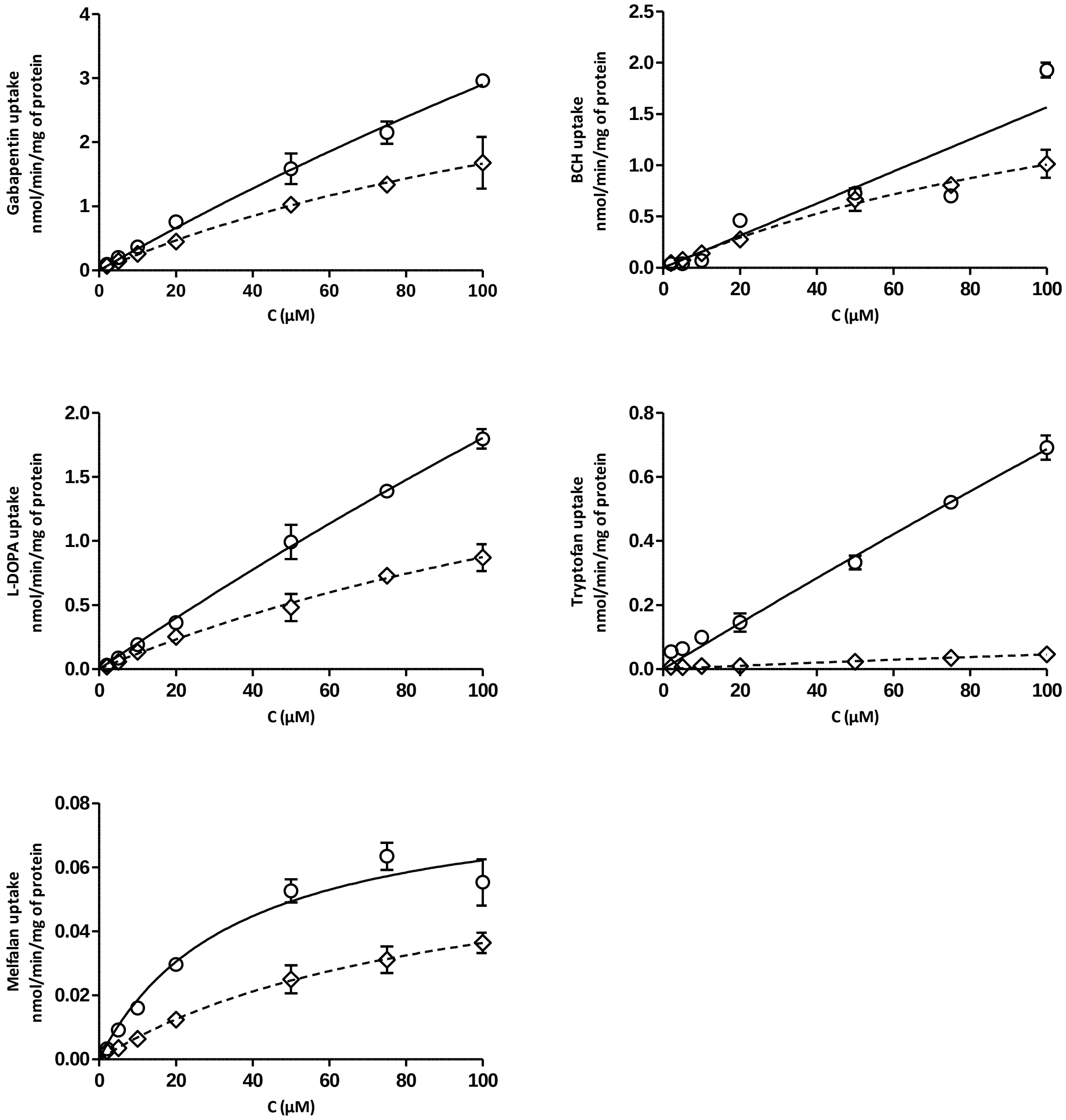

Figure S1. Uptake profiles of known substrates in HEK-hLAT1 cells (circle with solid line) and HEK-MOCK cells (square with dashed line). Please, note the different scales on the y-axis.

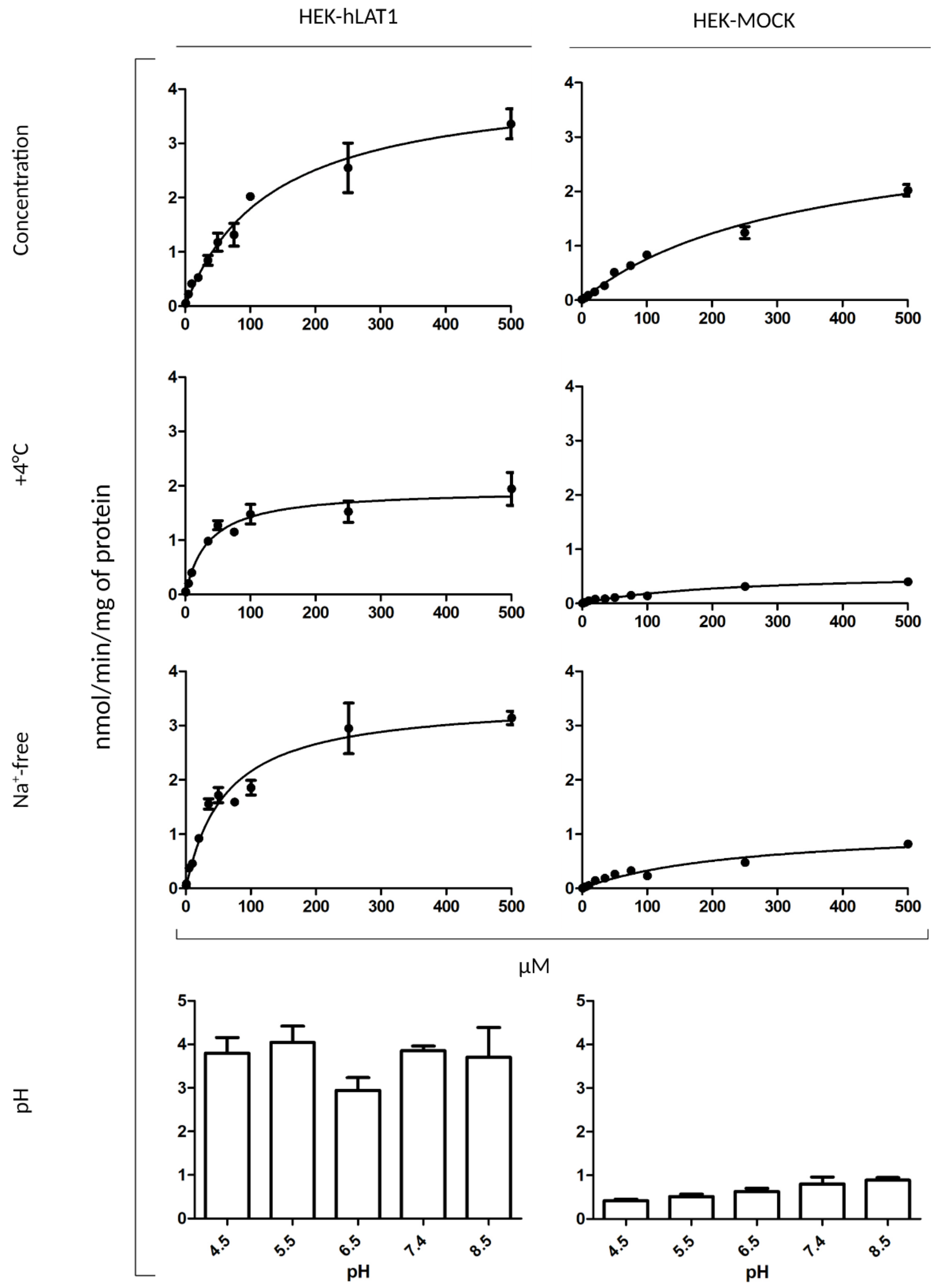

Figure S2. Characterization profiles for both HEK-hLAT1 and HEK-MOCK cells with [14C]-L-leucine as a substrate.

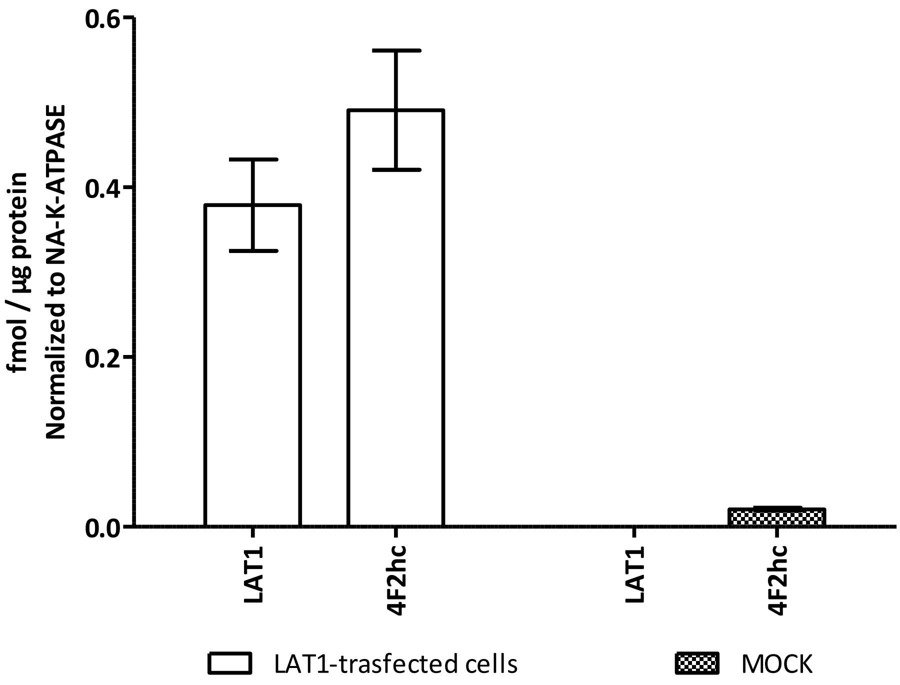

Figure S3. LAT1 and 4f2hc expression levels (fmol/µg of protein normalized to NA-K-ATPASE) in HEK-hLAT1 and HEK-MOCK cells.

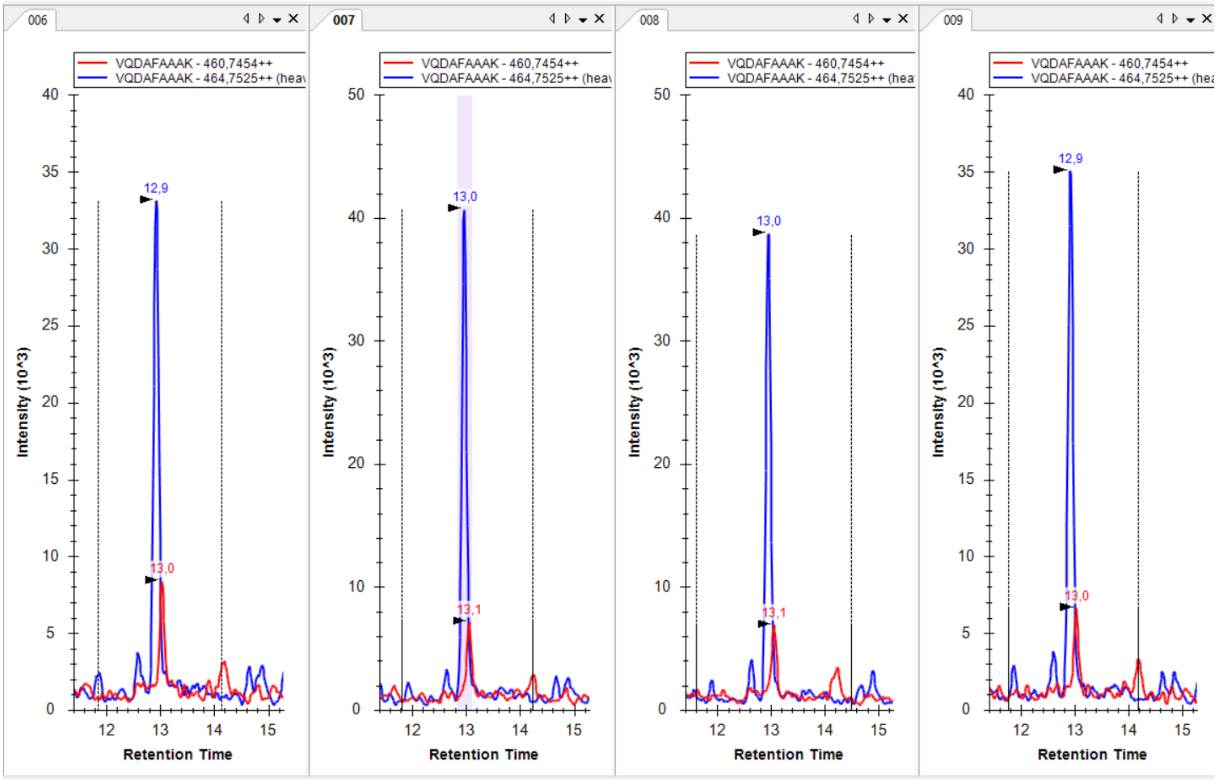

Figure S4. LAT1 expression in LAT1-transfected cells.

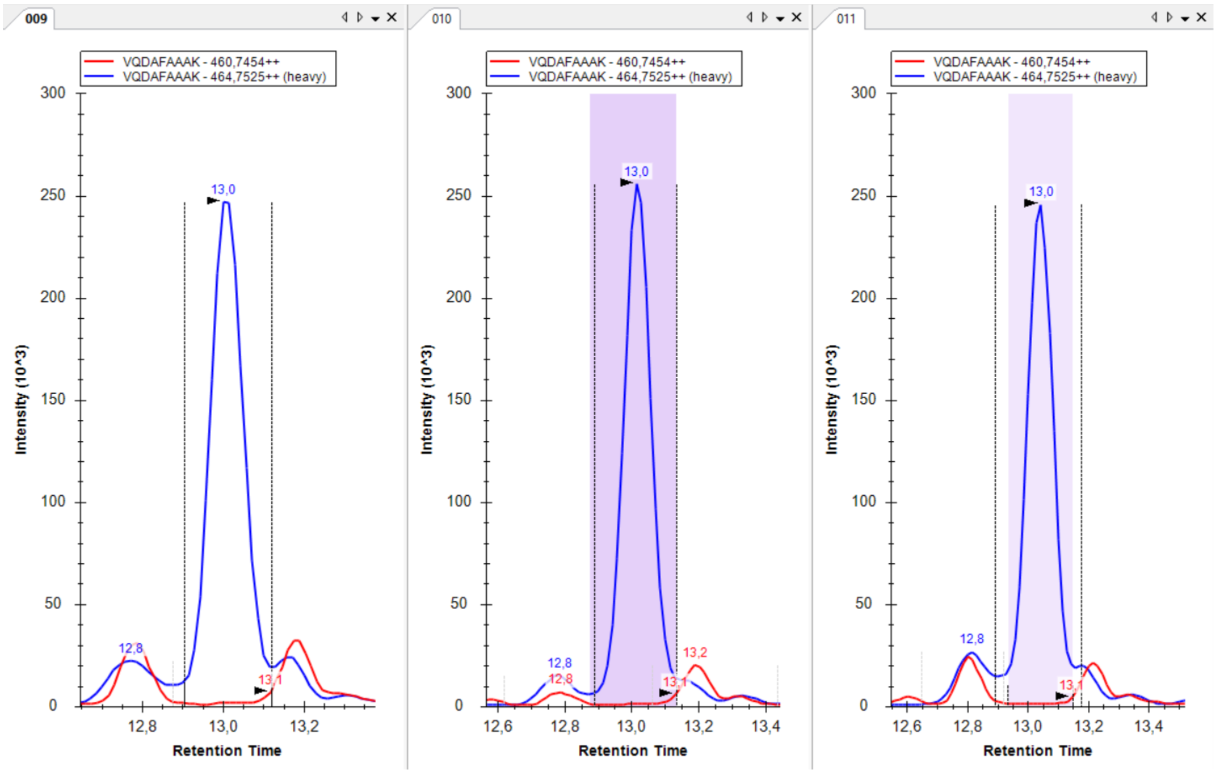

Figure S5. LAT1 expression in HEK-MOCK cells.

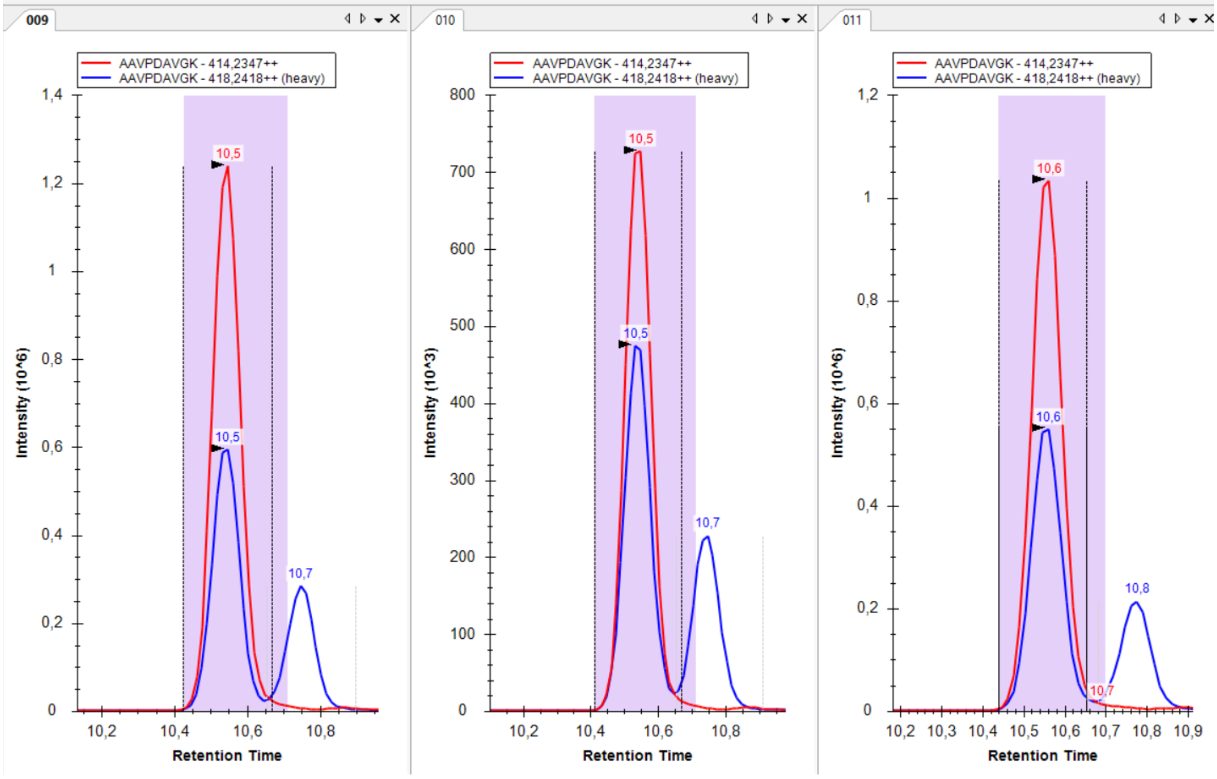

Figure S6. The Expression of Na+ K+ ATPase in HEK-MOCK cells.

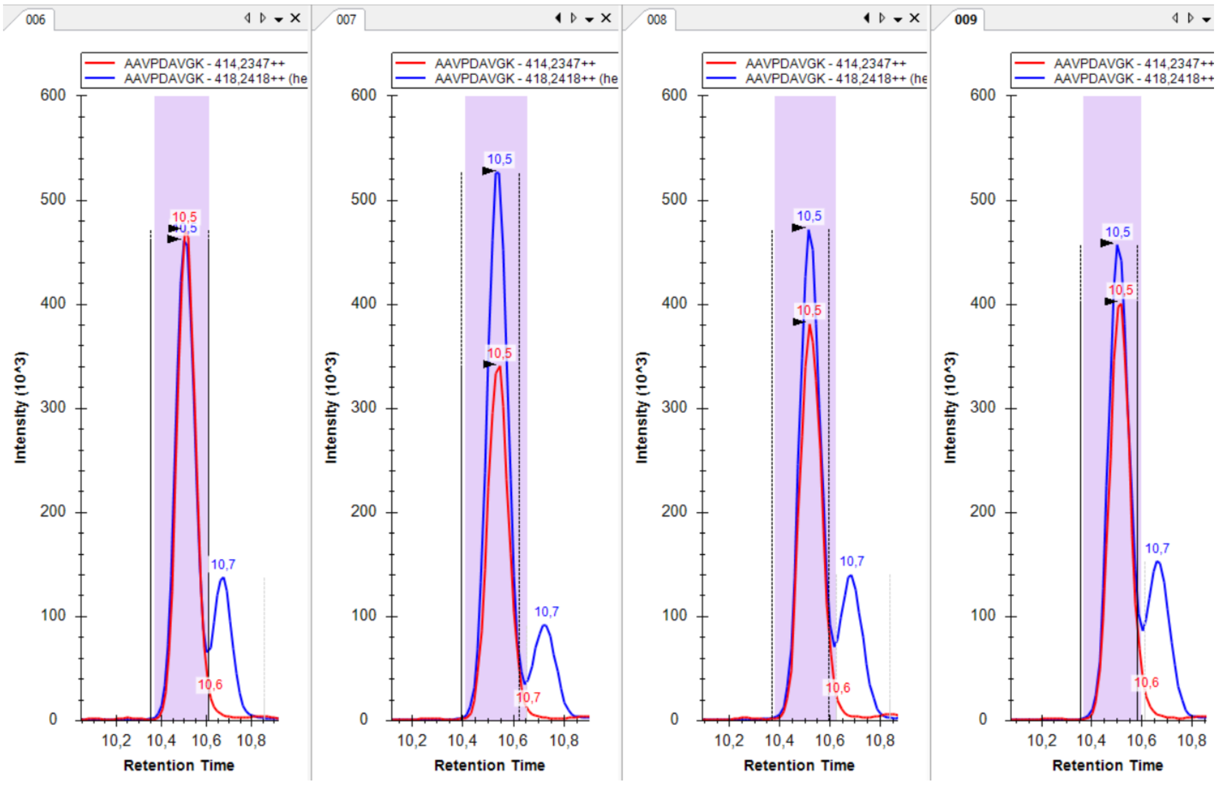

Figure S7. The Expression of Na+ K+ ATPase in LAT1-transfected cells.

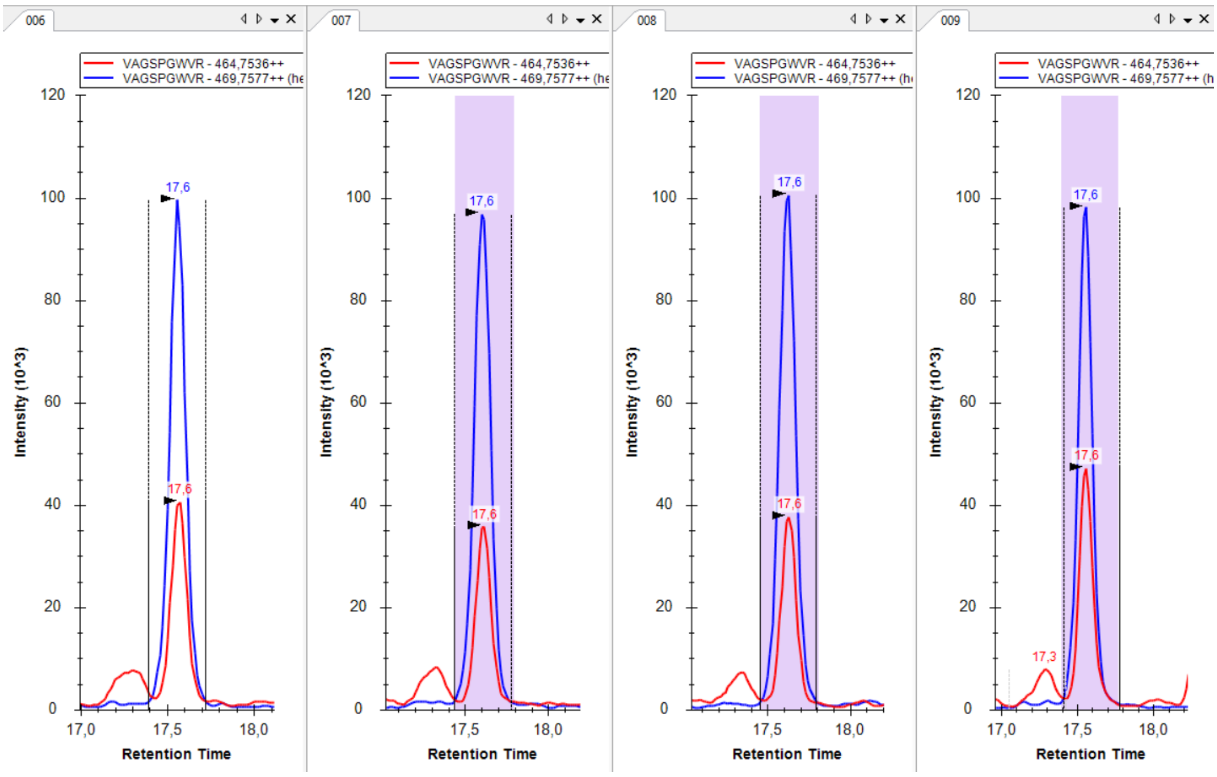

Figure S8. The Expression of 4f2hc in LAT1-transfected cells.

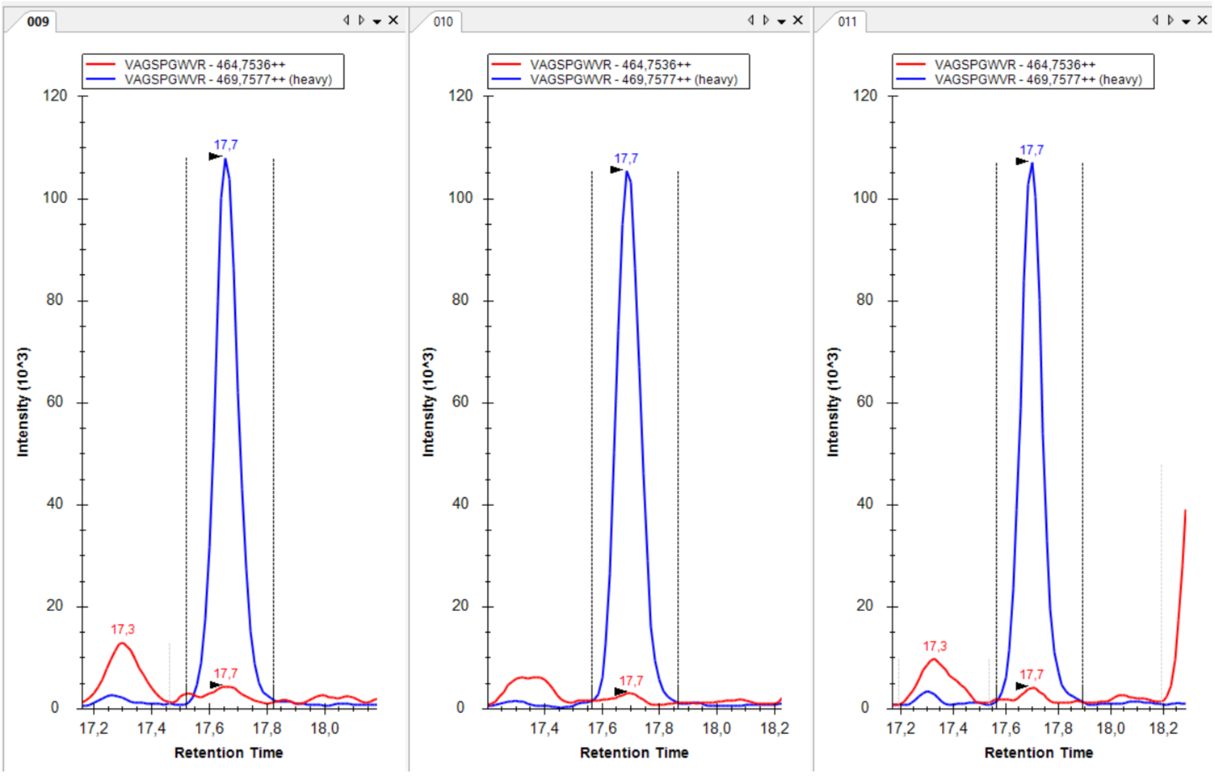

Figure S9. The Expression of 4f2hc in HEK-MOCK cells.

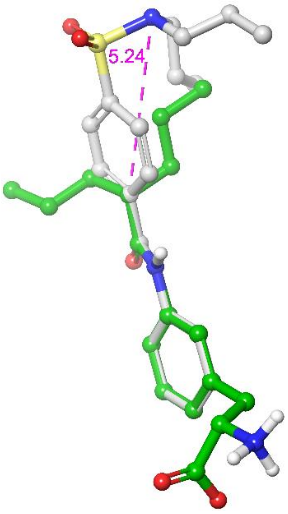

Figure S10. compound **4** (in green) aligned with **10**

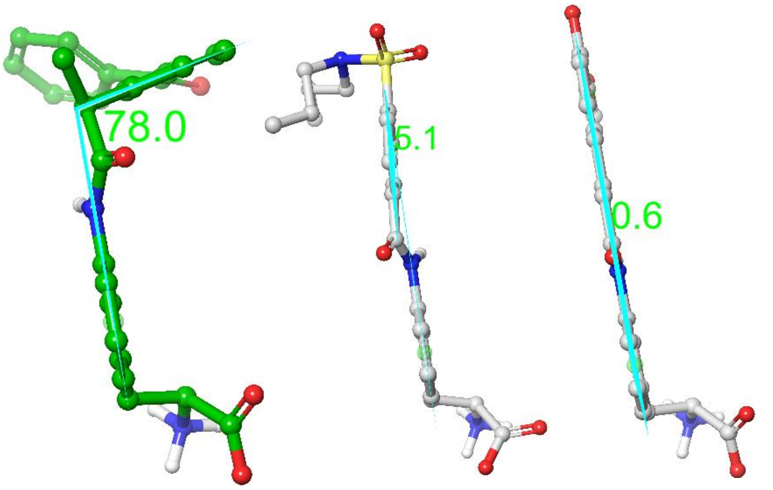

Figure S11. Angle between first and second ring of **5**, **10** and **9**

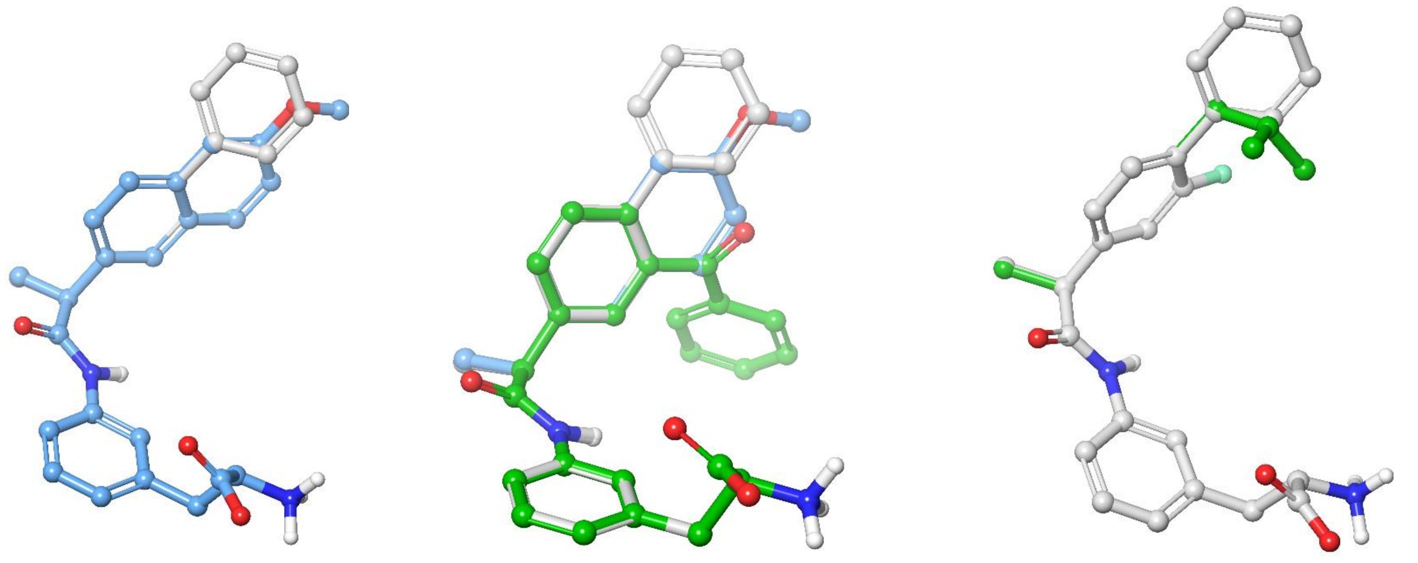

a b c

Figure S12. **(a)** compound **11** (in white) aligned with **12** (blue), **(b)** compound **11** (in white) and **12** (in blue) aligned with **5** (in green), **(c)** compound **11** in white aligned with **7** in green

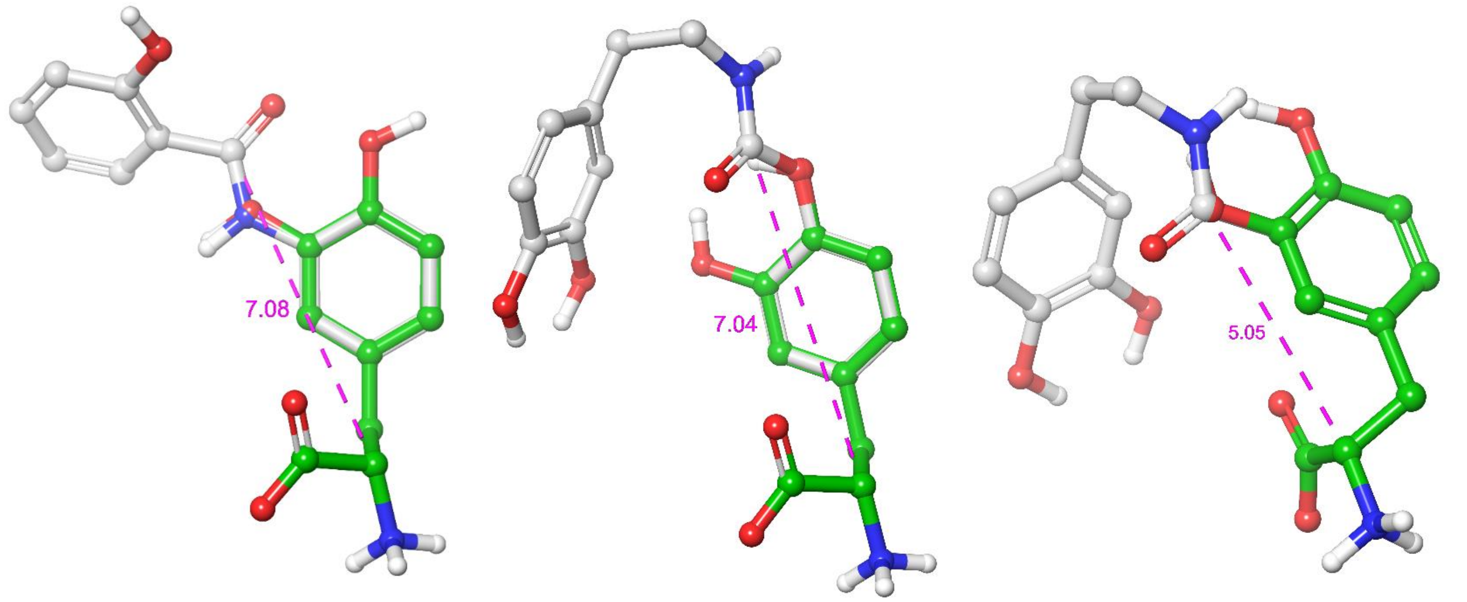

a b c

Figure S13. L-DOPA in green aligned with (a) compound **1**, (b) compound **2**, and (c) compound **3**
